# 884. Title: Factors Associated with Lack of Viral Suppression Among Women Living with HIV in the United States: An Integrative Review

**DOI:** 10.1093/ofid/ofab466.1079

**Published:** 2021-12-04

**Authors:** Titilola Labisi, Nada Fadul, Jason Coleman, Anthony Podany, Keyonna King

**Affiliations:** 1 University of Nebraska Medical Center, Omaha, Nebraska; 2 University of Nebraska Omaha, Omaha, Nebraska

## Abstract

**Background:**

Women account for 19% of new HIV cases in the United States (US). Transgender women are 49 times more likely than other groups to be diagnosed with HIV. HIV is one of the top ten causes of death among women between 25 to 44 years. Adherence to antiretroviral therapy (ART) and consequent viral suppression (VS) are keys to preventing sexual transmission, risk of drug resistance, and improving health outcomes. Hence, it is essential to identify factors behind VS in women living with HIV (WLWH).

**Methods:**

This review identified and synthesized peer-reviewed studies describing reasons for lack of VS among WLWH in the US. : Using the PRISMA model, we searched CINAHL, PubMed, Embase, Scopus, and PsycINFO, then selected US studies published from 2010 to April 2021. Studies that included men, non-adults, ongoing studies, and foreign studies were excluded. 1,359 studies were assessed and screened for duplicate and eligibility.

PRISMA Model

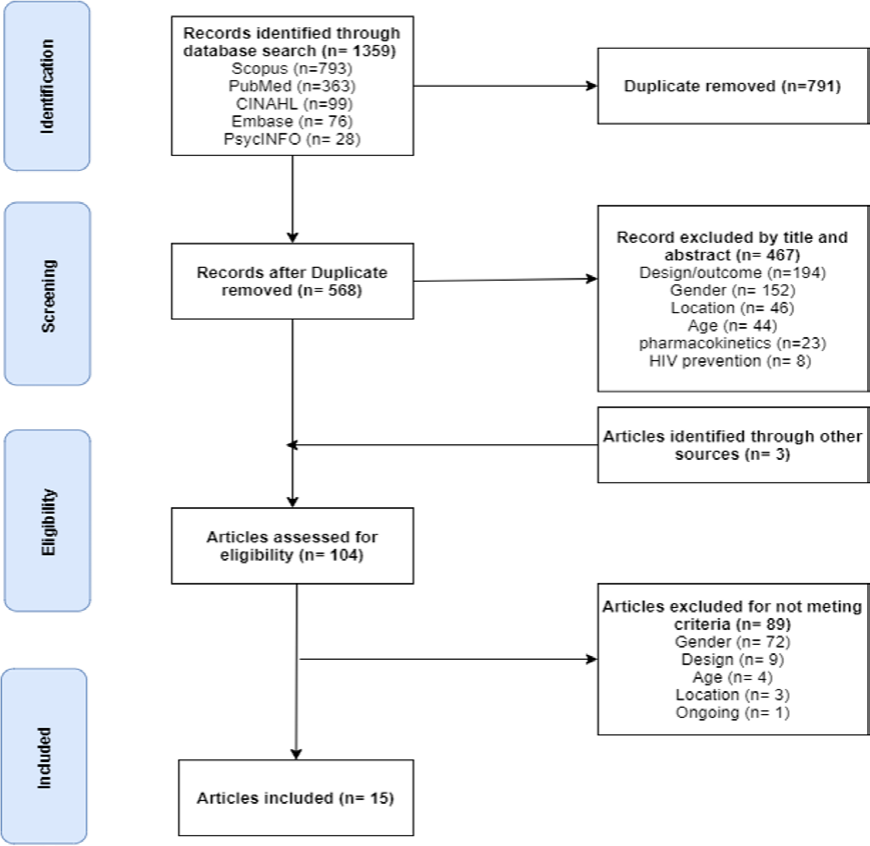

**Results:**

15 studies were eligible for review; 8 included all WLWH, 5 focused on pregnant WLWH, 1 included only African American WLWH and 1 included only transgender WLWH. Based on study participants and findings, results were divided into pregnancy and non-pregnancy-related factors. *Pregnancy-related factors:* Early ART initiation and group prenatal care improved care retention and VS. WLWH in cities were more likely to be virally suppressed at delivery than those in rural regions. Intimate partner violence (IPV) was associated with poor ART adherence and time to achieve stable VS. Also, being postpartum was associated with high viral load regardless of ART. *Non-pregnancy-related factors*: The most reported common factors were substance use and IPV. Other factors included social determinants of health, age, race, health insurance, income, number of pills, and regimen. Transgender-specific factors were stress, race, age, relationship, transphobic experiences, gender satisfaction, and adherence to hormone therapy.

**Conclusion:**

Substance use, income, mental health, health insurance, race, and ART regimen were the most common factors associated with VS in WLWH. There was paucity of data on transgender-specific VS factors. More research is needed to explore VS and treatment adherence amongWLWH, especially transgender women.

**Disclosures:**

**All Authors**: No reported disclosures

